# Association Between Different Patterns of Opioid and Benzodiazepine Use and Risks of Emergency Department Visits and Hospitalizations: A Retrospective Cohort Study

**DOI:** 10.3390/healthcare13162073

**Published:** 2025-08-21

**Authors:** Fang-Yu Su, Ming-Che Tsai, Yee-Yung Ng, Shiao-Chi Wu

**Affiliations:** 1 Institute of Health and Welfare Policy, School of Medicine, National Yang Ming Chiao Tung University, Taipei 112304, Taiwan; b5604119@gmail.com (F.-Y.S.); jazcacochi@gmail.com (M.-C.T.); 2 Department of Pharmacy, Linkou Chang Gung Memorial Hospital, Taoyuan 333423, Taiwan; 3 Department of Medicine, School of Medicine, Fu Jen Catholic University, New Taipei City 242062, Taiwan; yyngscwu12@gmail.com; 4 Department of Long-Term Care, College of Nursing, Asia University, Taichung 413305, Taiwan

**Keywords:** benzodiazepine, co-prescription, emergency department visits, hospitalizations, opioid users

## Abstract

**Background**: In 2016, the U.S. FDA warned against concurrent use of opioids and benzodiazepines (BZDs) due to risks of respiratory depression and death. However, limited data exist in Asian populations. **Methods**: Using the Chang Gung Research Database in Taiwan, we conducted a retrospective cohort study of 418,549 patients prescribed opioids between 2008 and 2018. Patients were categorized into four groups based on BZD use: opioid-only (PureO), past BZD use (PastB), continuous BZD use (ContiB), and newly initiated BZD use (NewB). Multivariate logistic regression was used to evaluate all-cause emergency department (ED) visits and hospitalizations during the one year follow-up following one year of co-use. **Results**: Compared with PureO, co-use groups had significantly higher odds of hospitalization (ContiB: aOR = 1.74; PastB: 1.54; NewB: 1.48) and ED visits (ContiB: 2.09; PastB: 2.04; NewB: 1.51). Elevated risks were also observed among older adults, and patients with depression, stroke or transient ischemic attack, chronic obstructive pulmonary disease, chronic kidney disease, as well as those with higher Charlson Comorbidity Index scores. **Conclusions**: Our findings support the need for cautious prescribing and individualized deprescribing strategies to reduce avoidable acute healthcare utilization.

## 1. Introduction

Chronic pain management commonly involves the use of nonsteroidal anti-inflammatory drugs (NSAIDs). However, patients with a history of gastrointestinal ulcers, renal insufficiency, or concurrent use of anticoagulants such as warfarin face an elevated risk of complications from NSAID therapy. In such high-risk populations, opioid analgesics are frequently considered alternative options for pain control [1].

Long-term opioid therapy is often associated with comorbid psychiatric and behavioral conditions, including anxiety, depression, insomnia, and substance use disorders [2,3]. Consequently, comprehensive pain management in this patient population necessitates a multidisciplinary approach that addresses both physical symptoms and psychological well-being. This complexity frequently leads to the co-prescription of psychotropic medications, including antidepressants, antiepileptics, antipsychotics, and sedative agents such as benzodiazepines (BZDs) [4].

Both opioids and BZDs are potent central nervous system (CNS) depressants, and their concurrent use poses significant risks. When co-administered, their respiratory depressant effects are not merely additive but may exhibit true synergistic interaction, particularly within critical brainstem structures. Multiple recent studies have underscored how this synergy—through the simultaneous activation of GABA-A and μ-opioid receptors—substantially impairs central respiratory drive, increasing the likelihood of life-threatening respiratory depression [5,6].

Although such prescribing patterns are prevalent in clinical practice, several guidelines, including those from the Australian Government, the United States Centers for Disease Control and Prevention (CDC), and the 2023 update of the American Geriatrics Society (AGS) Beers Criteria, have advised against the concurrent use of opioids and BZDs due to the heightened risk of respiratory depression and mortality [7,8,9,10]. Furthermore, the AGS Beers Criteria specifically highlight the increased risk of overdose and other adverse events, reinforcing the recommendation to avoid this combination.

Numerous studies conducted in the United States have investigated the consequences of this drug combination among diverse patient populations, such as military veterans, individuals living with HIV, residents of specific regions, privately insured cohorts, and patients diagnosed with post-traumatic stress disorder or chronic obstructive pulmonary disease (COPD) [11,12,13,14,15,16]. These studies consistently report an increased incidence of adverse clinical outcomes associated with opioid-BZD co-use.

In Taiwan, the use of opioid analgesics has demonstrated a steady upward trend over the past two decades. According to data from the National Health Research Institute (NHRI), the defined daily dose per million inhabitants per day (S-DDD/m/d) for opioids, including morphine, fentanyl, pethidine, codeine, and buprenorphine, increased from 631.2 in 2002 to 889.5 in 2014, representing a 41% rise in overall consumption [17]. Additionally, the total annual consumption of opioids nearly doubled between 2008 and 2018 [18]. Despite this increase, there is limited evidence concerning clinician awareness and prescribing behavior related to the risks of opioid and BZD co-prescription in Taiwan. Most existing studies are based on Western healthcare systems, and data from Asian contexts remain scarce.

The objective of this study was to assess the risk of serious adverse clinical events associated with high-risk medication combinations. Emergency department (ED) visits and hospital admissions were selected as the primary outcomes, as these endpoints reflect acute clinical deterioration and provide practical insight into the healthcare burden linked to such prescribing practices.

## 2. Methods

### 2.1. Data Set

This retrospective cohort study utilized data from the Chang Gung Research Database (CGRD), which compiles electronic medical records from the Chang Gung Memorial Hospital (CGMH) system. CGMH is currently the largest medical care system in Taiwan. It comprises three medical centers (Taipei, Linkou, and Kaohsiung branches), two regional hospitals (Keelung and Chiayi branches), and three district hospitals (Taoyuan, Fengshan, and Yunlin branches).

According to a previous study [19], inpatient and outpatient visits recorded in the CGRD between 1997 and 2010 accounted for 12.4% and 21.2%, respectively, of the cases included in the National Health Insurance Research Database (NHIRD). These figures highlight the CGMH system as the largest healthcare provider in Taiwan. The NHIRD covers more than 99.9% of the total population in Taiwan [20]. The CGRD contains a wide range of clinical information, including demographic data, inpatient and outpatient visit records, diagnostic codes, prescription details, and reports of imaging and functional examinations. The validity and accuracy of diagnostic codes in the CGRD have been confirmed in prior validation studies [21]. All data are stored at the Chang Gung Memorial Hospital Center. Only anonymized results that cannot be linked to any individual patient were accessed and analyzed in this study.

### 2.2. Study Design

The study period spanned from 1 January 2008 to 31 December 2018 to ascertain whether patients had used BZDs prior to the index date. This cohort included adult patients aged ≥20 years who were prescribed opioids within the Chang Gung Memorial Hospital (CGMH) system. Patients with a record of opioid dispensing between 1 January 2010 and 31 December 2015 were enrolled. Opioid prescriptions were identified using Anatomical Therapeutic Chemical (ATC) classification codes N02AA, N02AB, and N02AX.

**Figure 1:**
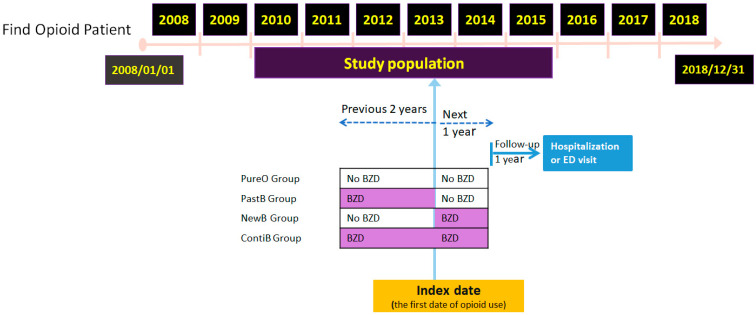
Scheme of the retrospective cohort study design. Abbreviation: BZD, benzodiazepines.

Patterns of opioid and BZD use during this period were examined using electronic medical records. A schematic overview of the retrospective cohort study is presented in Figure 1, which outlines patient inclusion, exposure group classification, index date assignment, and the one-year follow-up period for outcome assessment.

BZD use was defined based on ATC codes N03AE, N05BA, N05CD, and N05CF. Both inpatient and outpatient prescription records were included. Patients were excluded if they received only a single opioid prescription during the study period or if they were treated with methadone or buprenorphine for opioid dependence (ATC code N07BC). A complete list of ATC codes and corresponding drug names is provided in Supplementary File S1.

The index date was defined as the first day of opioid use. To capture temporal variations in BZD exposure, two periods were examined: the two years preceding the index date [22,23] and the one year following it [24]. Based on BZD use across these timeframes, patients were categorized into four mutually exclusive groups. Those with no BZD exposure in either period were classified as the PureO (opioid-only) group. Patients who used BZDs only prior to the index date were assigned to the PastB group, while those who used BZDs both before and after the index date were categorized as the ContiB group. Finally, patients who initiated BZD use only after the index date, without any prior exposure, were defined as the NewB group. This classification allowed for the assessment of how different patterns of BZD use may influence subsequent clinical outcomes.

This study was approved by the Institutional Review Board of the Chang Gung Medical Foundation (approval number: 201801306B0) and was conducted in accordance with relevant ethical guidelines.

### 2.3. Dependent Variables

This retrospective cohort study investigated two primary outcomes: all-cause hospital admissions and all-cause emergency department (ED) visits, which were analyzed separately. The index date was defined as the first day of opioid use, marking the start of the exposure period. Patients were required to maintain opioid use for one year (exposure period), and only those who survived until the end of this period were included in the subsequent analysis. Following the exposure period, eligible patients were followed for an additional one year (follow-up period), during which any all-cause hospital admission or ED visit was identified using visit type codes documented in the CGRD. This study design aimed to evaluate healthcare utilization and clinical risk factors associated with opioids and BZDs.

### 2.4. Independent Variable

The primary independent variable in this study was the pattern of BZD use among patients receiving opioids. To determine whether there was concurrent use of opioids and BZDs, we analyzed dispensing dates and days’ supply data from outpatient and inpatient pharmacy records and classified patients into four groups: ContiB, PastB, NewB, and PureO.

### 2.5. Control Variables

The demographics data included sex and age (five groups: 20–44, ≥45–64, ≥65–74, ≥75–84, and ≥85 years old). Comorbidities include cancer-related diagnoses (ICD-9-CM 140–239), depression (ICD-9-CM 300, 311), a history of stroke or transient ischemic attack (TIA) (ICD-9-CM 430–437), chronic obstructive pulmonary disease (COPD) (ICD-9-CM 490–496), and chronic kidney disease (CKD) (ICD-9-CM 580–587) [25]. The disease characteristics were defined according to the comorbidity status described in the Deyo–Romano Charlson Comorbidity Index (CCI) definition [26]. Comorbidities were identified based on outpatient and inpatient healthcare utilization within one year prior to the index date. The criteria for defining a comorbid condition required either at least two occurrences of primary or secondary diagnostic codes in outpatient claims or at least one occurrence of primary or secondary diagnostic codes in inpatient claims. Using the Deyo–Romano CCI definition, comorbidity scores were calculated and categorized as follows: no comorbidities (CCI = 0), mild comorbidities (CCI = 1), moderate comorbidities (CCI = 2), and severe comorbidities (CCI ≥ 3). This classification provides a systematic approach for assessing the comorbidity burden and its potential implications for the study population. The multicollinearity was investigated by the variance inflation factor (VIF) in regression analysis. When the VIF of each coefficient was less than 5, we presumed that the effect of correlation among the independent variables was not enough to distort the estimation.

### 2.6. Data Analysis

All statistical analyses were performed using the SAS System for Windows, version 9.4 (SAS Institute Inc., Cary, NC, USA). Descriptive statistics, including frequencies and percentages, were used to summarize the demographic characteristics of each combination group.

Two multivariable logistic regression models were constructed to evaluate the associations between adverse outcomes (dependent variable) and different opioid-BZD combination groups (primary independent variable). Model 1 adjusted for age, sex, and individual comorbidities. Model 2 included the same covariates as Model 1 but replaced individual comorbidities with the CCI score to account for overall comorbidity burden.

A two-sided *p*-value of less than 0.05 was considered statistically significant. To examine whether the associations between BZD use patterns and outcomes were consistent across subgroups (sex, age group, comorbidity type, and CCI score), stratified analyses were performed. The Breslow–Day test for homogeneity of odds ratios was used to evaluate whether the ORs across subgroups were statistically homogeneous.

## 3. Results

### 3.1. Demographics and Comorbidities of Opioid Users with and Without Benzodiazepine Use

According to Table 1, there were 418,549 patients prescribed opioids. Of these, 264,789 (63.26%) were in the PureO group, 68,845 (16.45%) in the NewB group, 53,016 (12.67%) in the PastB group, and 31,899 (7.62%) in the ContiB group. The proportion of females was slightly higher than males in both the PureO (63.51% vs. 62.99%) and ContiB (7.78% vs. 7.44%) groups. Notably, younger patients aged 20–44 were more likely to belong to the PureO group (80.21%) compared to older age groups (45–64: 59.64%, 65–74: 52.09%, 75–84: 48.75%, ≥85: 50.45%). Regarding comorbidities, patients with cancer were predominantly found in the PureO group (57.17%), while lower proportions were observed for those with depression (15.89%), stroke or TIA (33.03%), COPD (38.77%), and CKD (35.78%). Additionally, patients with higher CCI scores were more commonly observed in the ContiB group, suggesting a greater comorbidity burden in this subgroup.

### 3.2. Hospitalization Rate Among Patients in the Different Groups After 1-Year Follow-Up Study

As shown in Table 1, the one-year all-cause hospitalization rate among male patients was significantly higher than that of female patients across all groups (10.95% vs. 9.24% in PureO group, 16.66% vs. 14.38% in NewB, 18.93% vs. 16.45% in PastB, and 20.19% vs. 17.59% in ContiB, *p* < 0.001). Age group was a significant factor in hospitalization rates, with a notable increase in hospitalization rates observed with advancing age (*p* < 0.001). Furthermore, a significant proportion of hospitalizations were associated with individuals with comorbidities. The proportions of all-cause hospitalizations in each group demonstrated a clear correlation with the escalation of the comorbidity index (*p* < 0.001).

### 3.3. Emergency Department Visit Rate Among Patients in the Different Groups After 1-Year Follow-Up Study

As shown in Table 2, one-year all-cause ED visit rates were consistently higher in male patients compared to female patients across all groups: 10.86% vs. 8.92% in the PureO group, 15.97% vs. 14.80% in the NewB group, 23.56% vs. 22.25% in the PreB group, and 22.56% vs. 21.14% in the ContiB group (*p* < 0.001). Age was a significant determinant of ED utilization, with rates increasing progressively among patients aged 45 years and older (*p* < 0.001). Additionally, comorbid conditions were strongly associated with higher ED visit rates. A clear positive correlation was observed between all-cause ED visits and increasing levels of comorbidity, as measured by the comorbidity index (*p* < 0.001)

### 3.4. Odds Ratio of Hospitalizations in Each Group After 1-Year Follow-Up Study

As shown in Table 3, the results of the univariate and multivariable logistic regression analysis revealed that, compared to the PureO group (reference), all other groups with BZD use had significantly higher risks of one-year all-cause hospitalization. The risk factors for groups have the same ordinal relation in both models (i.e., NewB < PastB < ContiB), so there is minimal confounding with respect to how one adjusts for comorbidities. In Model 1, after control for comorbidities and other covariates, the ContiB group exhibited the highest adjusted odds ratio (aOR = 1.74; 95% CI: 1.69–1.80), followed by the PastB group (aOR = 1.54; 95% CI: 1.50–1.59) and the NewB group (aOR = 1.48; 95% CI: 1.44–1.51) (all *p* < 0.001). In Model 2, after control for CCI and other covariates, the ContiB group exhibited the highest adjusted odds ratio (aOR = 1.55; 95% CI: 1.50–1.60), followed by the PastB group (aOR = 1.43; 95% CI: 1.39–1.46) and the NewB group (aOR = 1.31; 95% CI: 1.28–1.35) (all *p* < 0.001)

Being male is a risk factor in both models, and the aORs are very close. Crude odds ratio (cOR) = 1.19; 95% CI: 1.17–1.21; in Model 1, aOR = 1.17; 95% CI: 1.14–1.19; *p* < 0.001; in Model 2, aOR = 1.09; 95% CI: 1.07–1.11; *p* < 0.001.

Risk escalates with increasing age; however, it does so faster in Model 1 than in Model 2. This indicates that concurrent comorbidities are a significant driver of hospitalization risk, and in Model 1, they confound the risk associated with increasing age. In Model 2, they are accounted for explicitly, so age itself appears as a weaker risk factor, but there is still an increasing risk with advancing age regardless. Compared with those aged 20–44 years, age was also associated with higher hospitalization risk, with a cOR of 1.29 for individuals aged 45–64 years and 1.91 for those aged ≥85 years. In Model 1, after controlling for comorbidities and other covariates, the aOR was 1.06 for those aged 45–64 years and 1.36 for those aged ≥85 years. In Model 2, after controlling for CCI and other covariates, the aOR was 0.91 for those aged 45–64 years, 1.05 for those aged 65–74 years, and 1.11 for those aged ≥85 years.

Regarding comorbidities, the risk of hospitalization was elevated in patients with CKD (aOR = 1.64; 95% CI: 1.59–1.70), a history of stroke or TIA (aOR = 1.37; 95% CI: 1.32–1.42), cancer (aOR = 1.35; 95% CI: 1.32–1.37), COPD (aOR = 1.32; 95% CI: 1.27–1.37), and depression (aOR = 1.16; 95% CI: 1.11–1.20).

### 3.5. Adjusted Odds Ratios for Emergency Department Visits After 1-Year Follow-Up

As shown in Table 4, except for the risk ordering of ED visits among groups (NewB < PastB ~ ContiB), the results were similar to those in Table 3. Multivariable logistic regression analysis demonstrated that, compared with the PureO reference group, all BZD co-use groups had significantly increased odds of all-cause ED visits within one year. In Model 1, the highest adjusted odds ratio was observed in the ContiB group (aOR = 2.09; 95% CI: 2.03–2.16), followed by the PastB group (aOR = 2.04; 95% CI: 1.99–2.10) and the NewB group (aOR = 1.51; 95% CI: 1.48–1.55), with all comparisons reaching statistical significance (*p* < 0.001). In Model 2, the highest adjusted odds ratio was observed in the PastB group (aOR = 2.07; 95% CI: 2.02–2.12), followed by the ContiB group (aOR = 1.97; 95% CI: 1.91–2.03) and the NewB group (aOR = 1.37; 95% CI: 1.34–1.41), with all comparisons reaching statistical significance (*p* < 0.001). When comparing PastB with ContiB in Model 1, their confidence intervals overlapped, so they can be treated as having equivalent risk, even though the aOR ordering appears to flip between Model 1 and Model 2.

Male patients had a slightly higher likelihood of ED visits compared to female patients (in Model 1, aOR = 1.11; 95% CI: 1.09–1.13; *p* < 0.001; in Model 2, aOR = 1.08; 95% CI: 1.06–1.10; *p* < 0.001). When stratified by age, the adjusted odds of ED visits varied across groups. In Model 1, using patients aged 20 to 44 years as the reference, those aged 45 to 64 years showed a modestly increased risk (aOR = 1.08; 95%CI: 1.05–1.11; *p* < 0.001). Patients aged 65 to 74 years had a slightly increased risk (aOR = 1.27; 95% CI: 1.23–1.30). A significantly higher risk was observed among patients aged 75 to 84 years (aOR = 1.56; 95% CI: 1.51–1.61; *p* < 0.001) and those aged 85 years and older (aOR = 1.69; 95% CI: 1.62–1.76; *p* < 0.001). In Model 2, compared to patients aged 20 to 44 years, those aged 45 to 64 years showed a modestly reduced risk (aOR = 0.93; 95%CI: 0.91–0.96; *p* < 0.001). Patients aged 65 to 74 years had a risk similar to the reference group (aOR = 1.07; 95% CI: 1.04–1.10). Significantly elevated risks were observed among patients aged 75 to 84 years (aOR = 1.36; 95% CI: 1.31–1.40; *p* < 0.001) and those aged 85 years and older (aOR = 1.52; 95% CI: 1.46–1.59; *p* < 0.001).

With respect to comorbid conditions, CKD was associated with the greatest increase in ED visit risk (aOR = 1.84; 95% CI: 1.78–1.90), followed by COPD (aOR = 1.49; 95% CI: 1.44–1.54), a history of stroke or TIA (aOR = 1.43; 95% CI: 1.38–1.49), and depression (aOR = 1.40; 95% CI: 1.34–1.45). In contrast, patients with cancer had a lower likelihood of ED visits (aOR = 0.94; 95% CI: 0.92–0.96), suggesting a potentially protective association.

## 4. Discussion

Among the 418,549 patients prescribed opioids, 63.26% were categorized into the PureO group, 16.45% into the NewB group, 12.67% into the PastB group, and 7.62% into the ContiB group. These findings indicate that BZD prescriptions were generally guided by patients’ clinical conditions, with BZD therapy often being discontinued following clinical improvement. Consequently, only a small proportion of patients (7.62%) remained on continuous opioid and BZD therapy over time (Table 1).

The higher proportion of female than male in PurO group (53.08% versus 46.92%), PastB group (57.81% versus 42.19%) and ContiB group (54% versus 46%) (Table 1) in this study might be related to more sensitive and lower tolerance to pain, and more prevalent of anxiety disorders in female [27,28].

Although the previous literature showed that anxiety decreased steadily with age even without treatment, anxiety disorders did not last until old age in most cases. A high prevalence of stress, anxiety, and depression among patients with chronic disease(s) was approximately 68.7%, 51.1%, and 58.8%, respectively [29]. Older age inevitably increases ED visits and hospitalization risk for a number of chronic diseases [30,31]. High comorbidity is found among the anxiety disorders and between the anxiety disorders and other mental disorders, respectively [32]. Those previous reports supported the significantly higher proportion of patients aged ≥45 years compared with those <45 years (*p* < 0.001) in the ContiB group in this study.

The risks of hospitalizations and ED visits in the PastB, NewB, and ContiB groups were significantly higher than in the PurO group in this study, which aligned with previous studies of North Carolina Medicaid patients [13], Quintiles IMS claim plan non-elderly patients [11], and privately insured patients in the US [24]. Those results supported that the prescription of BZD in patients already using opioid pain killers [7,8,9] had potential risks of ED visits and hospitalizations, especially in elderly patients and patients with higher CCI scores.

Among the comorbid conditions analyzed, cancer was associated with an increased risk of hospitalization but a reduced risk of ED visits. Individuals undergoing cancer treatment often have scheduled outpatient visits or multi-day planned hospital admissions. The lower likelihood of unplanned ED visits among oncology patients may reflect more structured care plans, consistent follow-up schedules [33], and sustained engagement with the same healthcare system, which together may help minimize ED visits.

Moreover, depression was associated with substantially increased use of acute healthcare services. Patients with depression demonstrated significantly higher adjusted odds of all-cause hospitalizations (aOR = 1.16; 95% CI: 1.11–1.20) and ED visits (aOR = 1.40; 95% CI: 1.34–1.45). This association was more evident in patients receiving BZD prescriptions, particularly within the PastB, NewB, and ContiB groups, compared to those prescribed opioids alone.

Patients with stroke or TIA comorbidity also had elevated risks of all-cause hospital admissions (aOR = 1.37; 95% CI: 1.32–1.42) and ED visits (aOR = 1.43; 95% CI: 1.38–1.49). Stroke or TIA is a known clinical factor that increases opioid-related overdose risk [25]. Although BZDs may have short-term efficacy for anxiety, their potential risks of post-stroke mortality and recurrent stroke [34] may contribute to the observed increases in hospital admissions and ED visits. Therefore, the use of opioids and BZDs in patients with stroke or TIA warrants special caution.

After adjusting for CCI score in Model 1, patients with COPD had increased risks of all-cause hospital admissions (aOR = 1.32; 95% CI: 1.27–1.37) and ED visits (aOR = 1.49; 95% CI: 1.44–1.54), consistent with previous studies [35] recommending avoidance or close monitoring of concurrent opioid and sedative use in elderly COPD patients due to the elevated risk of respiratory events, including COPD exacerbation and respiratory depression leading to hospitalization or ED visits.

Similarly, CKD patients showed increased risks of all-cause hospital admissions (aOR = 1.64; 95% CI: 1.59–1.70) and ED visits (aOR = 1.84; 95% CI: 1.78–1.90). Although opioids and BZDs play important roles in CKD pharmacotherapy, they are also associated with multiple adverse outcomes [36]. Our findings confirm the increased risks of adverse events with their use in CKD patients.

For hospitalizations, the greater increase in risk with age observed in Model 1 suggests that concurrent comorbidities are significant contributors to hospitalization risk. In Model 1, individual comorbidities may partially confound the association between age and risk, whereas Model 2 explicitly accounts for overall comorbidity burden using CCI. Nevertheless, even after accounting for comorbidity burden in Model 2, the risk of hospitalization still increased with age, indicating that age independently contributes to hospitalization risk.

For ED visits, the slight change in risk ordering between PastB and ContiB across models likely reflects random variation rather than a meaningful difference, as their aORs largely overlap, indicating comparable risk. In addition, the increase in risk with age was even more pronounced for ED visits than for hospitalizations, despite adjustment for concurrent comorbidities. This suggests that factors beyond comorbidity burden, such as frailty or functional decline associated with older age, may partially drive the increased likelihood of ED visits.

Our findings are consistent with those of Yarborough et al. [37], who reported that patients with anxiety disorders were significantly more likely to receive concomitant prescriptions for opioids and BZDs (aOR = 4.71; 95% CI: 2.67–8.32; *p* < 0.001), and that such co-prescription was associated with an elevated risk of ED visits (relative risk = 1.66; 95% CI: 1.08–2.53; *p* = 0.0194). Together, these results suggest that mental health conditions such as depression and anxiety increase the likelihood of central nervous system depressant co-use and subsequent acute care utilization. These findings underscore the importance of comprehensive mental health assessment and cautious medication management in patients with chronic pain, particularly those on long-term opioid therapy.

The final point pertains to the 2023 update of the AGS Beers Criteria, which identifies the concurrent use of opioids and BZDs as potentially inappropriate for adults aged 65 years and older due to the increased risk of adverse outcomes [10]. Our findings corroborate these recommendations, demonstrating that older adults receiving concomitant opioid-BZD therapy are more likely to experience increased utilization of emergency department services and hospital admissions.

Nonetheless, recent studies have raised important concerns regarding the potential harms of inappropriate or non-individualized deprescribing of BZDs. Maust et al. reported that discontinuing BZDs in patients maintained on long-term, stable regimens was associated with increased risks of mortality and other adverse outcomes [38]. In response to these findings, the American Society of Addiction Medicine (ASAM) released updated clinical guidelines in 2025, explicitly advising against inappropriate discontinuation or automatic tapering of BZDs without thorough clinical evaluation and individualized treatment planning [39]. Therefore, deprescribing decisions should be made with careful clinical judgment and involve shared decision making with patients to ensure both safety and treatment continuity.

In summary, although BZDs carry recognized risks, their therapeutic role remains relevant in appropriately selected patients. Decisions regarding deprescribing should be made on an individual basis, with a careful assessment of the benefit-risk balance, particularly in older adults and those on chronic therapy. Abrupt discontinuation may lead to unintended harm; therefore, clinical discretion and shared decision making are essential for ensuring safe and effective BZD management. Future research should focus on evaluating deprescribing interventions and developing patient-specific risk stratification strategies to guide clinical decision making.

### Limitations and Strengths

This study has several limitations. First, the Chang Gung Research Database (CGRD) provides information on prescription duration but does not capture whether patients actually took the prescribed medications [25]. As a result, the influence of dosage and medication adherence on adverse outcomes, such as ED visits and hospitalizations, could not be assessed in this analysis and should be addressed in future research. Second, the CGRD includes data from a single healthcare system; thus, medication use outside of the Chang Gung Memorial Hospital network could not be captured. This may have led to an underestimation of actual drug exposure and limits the generalizability of the findings to other healthcare settings. Third, the present analysis focused only on the two main classes of drugs—opioids and BZDs. Future research should further explore the effects of drug characteristics, such as duration of action (short-acting vs. long-acting) and dosage, to provide more comprehensive risk assessments.

Nevertheless, this study has important strengths. It draws from a large and representative sample of 418,549 patients within one of Taiwan’s largest healthcare systems, which supports the robustness and applicability of the findings to broader clinical settings. The results are consistent with international clinical guidelines, including those issued by the Centers for Disease Control and Prevention [7,8] and the 2023 American Geriatrics Society Beers Criteria [10], both of which recommend avoiding concurrent use of opioids and benzodiazepines, particularly in older adults. In addition, this study provides Taiwan-specific evidence that addresses a notable gap in the current literature. These findings offer meaningful implications for clinical practice and health policy, especially in regions where localized data have been limited compared to Western countries.

## 5. Conclusions

Patients receiving both opioids and benzodiazepines had a higher likelihood of emergency department (ED) visits and hospitalizations compared to those prescribed opioids alone, regardless of whether benzodiazepine use was past, continuous, or newly initiated. This increased risk was particularly evident among older adults with depression or higher CCI scores. Based on these findings, concurrent use of opioids and benzodiazepines should be avoided when possible, and alternative therapeutic options should be considered. Clinicians should remain vigilant in managing such combinations, as co-use may result in greater acute healthcare utilization. At the same time, decisions to taper or discontinue benzodiazepines must be carefully individualized to avoid adverse outcomes associated with inappropriate deprescribing.

## Figures and Tables

**Table 1:** Rates of hospitalization among individuals in the different groups after 1-year follow-up study.

Variable	All			PureO			PastB			NewB			ContiB			*p* ^a^
	N	%	*p*	N	%	*p*	N	%	*p*	N	%	*p*	N	%	*p*	
All	418,549	12.56		264,789	10.04		53,016	17.50		68,845	15.57		31,899	18.79		
Gender			<0.001			<0.001			<0.001			<0.001			<0.001	0.831
Male	197,256	13.58		124,250	10.95		22,366	18.93		35,965	16.66		14,675	20.19		
Female	221,293	11.65		140,539	9.24		30,650	16.45		32,880	14.38		17,224	17.59		
Age Group			<0.001			<0.001			<0.001			<0.001			<0.001	<0.001
20–44	131,481	9.63		105,455	8.48		7706	13.46		13,900	14.32		4420	15.52		
45–64	155,651	12.06		92,828	9.45		20,068	15.45		29,317	15.38		13,438	17.79		
65–74	64,064	15.32		33,374	12.73		11,316	19.43		12,677	15.95		6697	20.04		
75–84	49,809	16.80		24,281	14.06		10,188	21.14		9810	16.73		5530	21.01		
≥85	17,544	16.88		8851	13.63		3738	21.03		3141	17.77		1814	22.71		
Comorbidity																
Cancer	110,470	15.40	<0.001	63,157	12.33	<0.001	14,141	19.45	<0.001	22,971	18.81	<0.001	10,201	21.13	<0.001	<0.001
Depression	18,736	18.26	<0.001	2978	11.32	0.020	10,253	19.67	<0.001	1923	17.21	0.044	3582	20.58	0.004	0.667
Past history of stroke or TIA	19,959	21.03	<0.001	6593	16.91	<0.001	6883	23.51	<0.001	3418	19.89	<0.001	3065	25.58	<0.001	<0.001
COPD	20,203	20.32	<0.001	7833	15.84	<0.001	5785	23.99	<0.001	3713	20.87	<0.001	2872	24.44	<0.001	0.003
CKD	22,273	22.78	<0.001	7969	18.53	<0.001	6621	26.11	<0.001	4517	22.03	<0.001	3166	27.54	<0.001	<0.001
CCI score			<0.001			<0.001			<0.001			<0.001			<0.001	<0.001
CCI = 0	250,012	8.58		188,002	7.78		19,152	10.78		31,301	10.76		11,557	12.17		
CCI = 1	53,389	13.19		27,569	10.90		10,541	15.83		9654	14.41		5625	17.37		
CCI = 2	50,555	18.11		24,693	15.17		8845	20.79		11,469	20.52		5548	21.92		
CCI ≥ 3	64,593	23.11		24,525	21.28		14,478	25.59		16,421	21.98		9169	26.10		

Abbreviations: CKD, chronic kidney disease; COPD, chronic obstructive pulmonary disease; TIA, transient ischemic attack. Note: the distribution among groups was analyzed by x^2^ test. Note: *p* ^a^, Breslow–Day test.

**Table 2:** Rates of emergency department visits among individuals in the different groups after 1-year follow-up study.

Variable	All			PureO			PastB			NewB			ContiB			*p* ^a^
	N	%	*p*	N	%	*p*	N	%	*p*	N	%	*p*	N	%	*p*	
All	418,549	13.30		264,789	9.83		53,016	22.80		68,845	15.41		31,899	21.79		
Gender			<0.001			<0.001			0.001			<0.001			0.002	<0.001
Male	197,256	14.11		124,250	10.86		22,366	23.56		35,965	15.97		14,675	22.56		
Female	221,293	12.59		140,539	8.92		30,650	22.25		32,880	14.80		17,224	21.14		
Age Group			<0.001			<0.001			<0.001			<0.001			<0.001	<0.001
20–44	131,481	9.66		105,455	7.82		7706	20.23		13,900	14.22		4420	20.86		
45–64	155,651	12.32		92,828	9.04		20,068	20.06		29,317	14.02		13,438	19.67		
65–74	64,064	15.71		33,374	12.01		11,316	23.10		12,677	15.54		6697	21.97		
75–84	49,809	19.93		24,281	15.75		10,188	27.68		9810	19.18		5530	25.35		
≥85	17,544	21.74		8851	17.58		3738	28.68		3141	21.43		1814	28.28		
Comorbidity																
Cancer	110,470	13.24	0.467	63,157	9.85	0.838	14,141	20.72	<0.001	22,971	15.12	0.131	10,201	19.62	<0.001	<0.001
Depression	18,736	24.86	<0.001	2978	15.14	<0.001	10,253	27.86	<0.001	1923	21.63	<0.001	3582	26.07	<0.001	0.002
Past history of stroke or TIA	19,959	25.57	<0.001	6593	20.08	<0.001	6883	30.15	<0.001	3418	23.08	<0.001	3065	29.89	<0.001	<0.001
COPD	20,203	24.85	<0.001	7833	19.28	<0.001	5785	31.03	<0.001	3713	23.92	<0.001	2872	28.83	<0.001	<0.001
CKD	22,273	27.59	<0.001	7969	21.37	<0.001	6621	33.35	<0.001	4517	25.73	<0.001	3166	33.86	<0.001	<0.001
CCI score			<0.001			<0.001			<0.001			<0.001			<0.001	<0.001
CCI = 0	250,012	9.11		188,002	7.64		19,152	15.95		31,301	11.12		11,557	16.30		
CCI = 1	53,389	17.10		27,569	13.21		10,541	23.53		9654	18.14		5625	22.40		
CCI = 2	50,555	18.15		24,693	13.94		8845	25.92		11,469	18.15		5548	24.48		
CCI ≥ 3	64,593	22.61		24,525	18.72		14,478	29.44		16,421	20.08		9169	26.71		

Abbreviations: CKD, chronic kidney disease; COPD, chronic obstructive pulmonary disease; TIA, transient ischemic attack. Note: the distribution among groups was analyzed by x^2^ test. Note: *p* ^a^, Breslow–Day test.

**Table 3:** The odds ratios of hospitalization in each group after 1-year follow-up study.

Variable	Univariate			Model 1			Model 2		
	cOR	95% CI	*p*	aOR	95% CI	*p*	aOR	95% CI	*p*
Group (ref: PureO)									
PastB	1.90	(1.85–1.95)	<0.001	1.54	(1.50–1.59)	<0.001	1.43	(1.39–1.46)	<0.001
NewB	1.65	(1.61–1.69)	<0.001	1.48	(1.44–1.51)	<0.001	1.31	(1.28–1.35)	<0.001
ContiB	2.07	(2.01–2.14)	<0.001	1.74	(1.69–1.80)	<0.001	1.55	(1.50–1.60)	<0.001
Gender (ref: Female)									
Male	1.19	(1.17–1.21)	<0.001	1.17	(1.14–1.19)	<0.001	1.09	(1.07–1.11)	<0.001
Age Group (ref: 20–44)									
45–64	1.29	(1.26–1.32)	<0.001	1.06	(1.04–1.09)	<0.001	0.91	(0.89–0.94)	<0.001
65–74	1.70	(1.65–1.75)	<0.001	1.30	(1.26–1.34)	<0.001	1.05	(1.01–1.08)	0.004
75–84	1.90	(1.84–1.95)	<0.001	1.37	(1.33–1.42)	<0.001	1.10	(1.07–1.14)	<0.001
≥85	1.91	(1.83–1.99)	<0.001	1.36	(1.30–1.42)	<0.001	1.11	(1.06–1.16)	<0.001
Comorbidity									
Cancer	1.40	(1.37–1.42)	<0.001	1.35	(1.32–1.37)	<0.001			
Depression	1.59	(1.53–1.66)	<0.001	1.16	(1.11–1.20)	<0.001			
Past history of stroke or TIA	1.93	(1.86–2.00)	<0.001	1.37	(1.32–1.42)	<0.001			
COPD	1.84	(1.78–1.91)	<0.001	1.32	(1.27–1.37)	<0.001			
CKD	2.17	(2.10–2.24)	<0.001	1.64	(1.59–1.70)	<0.001			
CCI score (ref: CCI = 0)									
CCI = 1	1.62	(1.57–1.67)	<0.001				1.46	(1.42–1.50)	<0.001
CCI = 2	2.36	(2.29–2.42)	<0.001				2.12	(2.06–2.18)	<0.001
CCI ≥ 3	3.20	(3.13–3.28)	<0.001				2.72	(2.65–2.79)	<0.001

Multivariate logistic regression. Abbreviations: CKD, chronic kidney disease; COPD, chronic obstructive pulmonary disease; TIA, transient ischemic attack. Model 1: adjusted for age, sex, and individual comorbidities. Model 2: adjusted for age, sex, and CCI score.

**Table 4:** The odds ratios of emergency department visits in each group after 1-year follow-up study.

Variable	Univariate			Model 1			Model 2		
	cOR	95%CI	*p*	aOR	95% CI	*p*	aOR	95% CI	*p*
Group (ref: PureO)									
PastB	2.71	(2.65–2.78)	<0.001	2.04	(1.99–2.10)	<0.001	2.07	(2.02–2.12)	<0.001
NewB	1.67	(1.63–1.71)	<0.001	1.51	(1.48–1.55)	<0.001	1.37	(1.34–1.41)	<0.001
ContiB	2.56	(2.48–2.63)	<0.001	2.09	(2.03–2.16)	<0.001	1.97	(1.91–2.03)	<0.001
Gender (ref: Female)									
Male	1.14	(1.12–1.16)	<0.001	1.11	(1.09–1.13)	<0.001	1.08	(1.06–1.10)	<0.001
Age Group (ref: 20–44)									
45–64	1.31	(1.28–1.35)	<0.001	1.08	(1.05–1.11)	<0.001	0.93	(0.91–0.96)	<0.001
65–74	1.74	(1.69–1.79)	<0.001	1.27	(1.23–1.30)	<0.001	1.07	(1.04–1.10)	<0.001
75–84	2.33	(2.26–2.40)	<0.001	1.56	(1.51–1.61)	<0.001	1.36	(1.31–1.40)	<0.001
≥85	2.60	(2.49–2.70)	<0.001	1.69	(1.62–1.76)	<0.001	1.52	(1.46–1.59)	<0.001
Comorbidity									
Cancer	0.99	(0.97–1.01)	0.467	0.94	(0.92–0.96)	<0.001			
Depression	2.26	(2.19–2.34)	<0.001	1.40	(1.34–1.45)	<0.001			
Past history of stroke or TIA	2.36	(2.29–2.44)	<0.001	1.43	(1.38–1.49)	<0.001			
COPD	2.27	(2.20–2.35)	<0.001	1.49	(1.44–1.54)	<0.001			
CKD	2.67	(2.59–2.75)	<0.001	1.84	(1.78–1.90)	<0.001			
CCI score (ref: CCI = 0)									
CCI = 1	2.06	(2.01–2.11)	<0.001				1.68	(1.63–1.72)	<0.001
CCI = 2	2.21	(2.15–2.27)	<0.001				1.80	(1.75–1.86)	<0.001
CCI ≥ 3	2.92	(2.85–2.98)	<0.001				2.16	(2.10–2.21)	<0.001

Multivariate logistic regression. Abbreviations: CKD, chronic kidney disease; COPD, chronic obstructive pulmonary disease; TIA, transient ischemic attack. Model 1: adjusted for age, sex, and individual comorbidities. Model 2: adjusted for age, sex, and CCI score.

## Data Availability

Restrictions apply to the availability of these data. The data were obtained from the Chang Gung Research Database (CGRD) and are available from the corresponding author with the permission of the CGRD.

## Supplementary Materials

The following supporting information can be downloaded at: https://www.mdpi.com/article/10.3390/healthcare13162073/s1, File S1: Anatomical Therapeutic Chemical [ATC] code.

## Abbreviations

The following abbreviations are used in this manuscript:

BZD	Benzodiazepine
CKD	Chronic kidney disease
COPD	Chronic obstructive pulmonary disease
TIA	Transient ischemic attack

## References

[1] Freynhagen R., Geisslinger G., Schug S.A. (2013). Opioids for chronic non-cancer pain. Br. Med. J..

[2] Vogt S., Pfau G., Vielhaber S., Haghikia A., Hachenberg T., Brinkers M. (2023). Long-term opioid therapy and mental health comorbidity in patients with chronic pain. Pain Med..

[3] Leung J., Santo T., Colledge-Frisby S., Mekonen T., Thomson K., Degenhardt L., Connor J.P., Hall W., Stjepanovic D. (2022). Mood and Anxiety Symptoms in Persons Taking Prescription Opioids: A Systematic Review with Meta-Analyses of Longitudinal Studies. Pain Med..

[4] Zin C.S., Ismail F. (2017). Co-prescription of opioids with benzodiazepine and other co-medications among opioid users: Differential in opioid doses. J. Pain Res..

[5] Lynch N., Lima J.D., Spinieli R.L., Kaur S. (2023). Opioids, sleep, analgesia and respiratory depression: Their convergence on Mu (mu)-opioid receptors in the parabrachial area. Front. Neurosci..

[6] Boon M., van Dorp E., Broens S., Overdyk F. (2020). Combining opioids and benzodiazepines: Effects on mortality and severe adverse respiratory events. Ann. Palliat. Med..

[7] Dowell D. (2022). CDC clinical practice guideline for prescribing opioids for pain—United States, 2022. MMWR. Recomm. Rep..

[8] Dowell D., Haegerich T.M., Chou R. (2016). CDC Guideline for Prescribing Opioids for Chronic Pain—United States, 2016. J. Am. Med. Assoc..

[9] Molbournr TGLec. Analgesia Guideline 2013. https://tgldcdp.tg.org.au/guideLine?guidelinePage=Analgesic&frompage=etgcomplete.

[10] By the 2023 American Geriatrics Society Beers Criteria^®^ Update Expert Panel (2023). American Geriatrics Society 2023 updated AGS Beers Criteria(R) for potentially inappropriate medication use in older adults. J. Am. Geriatr. Soc..

[11] Chang H.-Y., Kharrazi H., Bodycombe D., Weiner J.P., Alexander G.C. (2018). Healthcare costs and utilization associated with high-risk prescription opioid use: A retrospective cohort study. BMC Med..

[12] Dasgupta N., Funk M.J., Proescholdbell S., Hirsch A., Ribisl K.M., Marshall S. (2016). Cohort study of the impact of high-dose opioid analgesics on overdose mortality. Pain Med..

[13] Hung A., Bush C., Greiner M., Campbell H., Hammill B., Maciejewski M.L., McKethan A. (2020). Risk Factors and Outcomes of Opioid Users with and Without Concurrent Benzodiazepine Use in the North Carolina Medicaid Population. J. Manag. Care Spec. Pharm..

[14] Parent S., Nolan S., Fairbairn N., Ye M., Wu A., Montaner J., Barrios R., Ti L., Daly P., Gilbert M. (2019). Correlates of opioid and benzodiazepine co-prescription among people living with HIV in British Columbia, Canada: A population-level cohort study. Int. J. Drug Policy.

[15] Jiang H., Zhang X., Zhang J., Liang J., Wang L. (2024). Association Between Opioid and Benzodiazepine Use and All-Cause Mortality in Individuals with Chronic Obstructive Pulmonary Disease: A Prospective Cohort Study. Int. J. Chronic Obstr. Pulm. Dis..

[16] Sullivan M. (2018). Dangerously numb: Opioids, benzodiazepines, chronic pain, and posttraumatic stress disorder. Pain.

[17] Kang K.H., Kuo L.F., Cheng I.C., Chang C.S., Tsay W.I. (2017). Trends in major opioid analgesic consumption in Taiwan, 2002–2014. J. Formos. Med. Assoc..

[18] Wang J.-J., Teng S.-F., Chu Y.-R., Chu C.-C., Ho C.-H., Chu L.-L. (2022). Evaluation of opioid consumption trends for pain in Taiwan and comparison with neighboring Asian countries. J. Food Drug Anal..

[19] Tsai M.S., Lin M.H., Lee C.P., Yang Y.H., Chen W.C., Chang G.H., Tsai Y.T., Chen P.C., Tsai Y.H. (2017). Chang Gung Research Database: A multi-institutional database consisting of original medical records. Biomed. J..

[20] Lin L.-Y., Warren-Gash C., Smeeth L., Chen P.-C. (2018). Data resource profile: The national health insurance research database (NHIRD). Epidemiol. Health.

[21] Shao S.C., Chan Y.Y., Kao Yang Y.H., Lin S.J., Hung M.J., Chien R.N., Lai C.C., Lai E.C. (2019). The Chang Gung Research Database-A multi-institutional electronic medical records database for real-world epidemiological studies in Taiwan. Pharmacoepidemiol. Drug Saf..

[22] Islam M.M. (2019). Pattern and probability of dispensing of prescription opioids and benzodiazepines among the new users in Australia: A retrospective cohort study. BMJ Open.

[23] Sung H.G., Li J., Nam J.H., Won D.Y., Choi B., Shin J.Y. (2019). Concurrent use of benzodiazepines, antidepressants, and opioid analgesics with zolpidem and risk for suicide: A case-control and case-crossover study. Soc. Psychiatry Psychiatr. Epidemiol..

[24] Sun E.C., Dixit A., Humphreys K., Darnall B.D., Baker L.C., Mackey S. (2017). Association between concurrent use of prescription opioids and benzodiazepines and overdose: Retrospective analysis. Br. Med. J..

[25] Hernandez I., He M., Brooks M.M., Zhang Y. (2018). Exposure-Response Association Between Concurrent Opioid and Benzodiazepine Use and Risk of Opioid-Related Overdose in Medicare Part D Beneficiaries. JAMA Netw. Open.

[26] Romano P.S., Roos L.L., Jollis J.G. (1993). Presentation adapting a clinical comorbidity index for use with ICD-9-CM administrative data: Differing perspectives. J. Clin. Epidemiol..

[27] McLean C.P., Asnaani A., Litz B.T., Hofmann S.G. (2011). Gender differences in anxiety disorders: Prevalence, course of illness, comorbidity and burden of illness. J. Psychiatr. Res..

[28] Failla M.D., Beach P.A., Atalla S., Dietrich M.S., Bruehl S., Cowan R.L., Monroe T.B. (2024). Gender differences in pain threshold, unpleasantness, and descending pain modulatory activation across the adult life span: A cross sectional study. J. Pain.

[29] M S., S M., Vadakkiniath I.J., A G. (2023). Prevalence and correlates of stress, anxiety, and depression in patients with chronic diseases: A cross-sectional study. Middle East. Curr. Psychiatry.

[30] Prince M.J., Wu F., Guo Y., Gutierrez Robledo L.M., O’Donnell M., Sullivan R., Yusuf S. (2015). The burden of disease in older people and implications for health policy and practice. Lancet.

[31] Kastner M., Cardoso R., Lai Y., Treister V., Hamid J.S., Hayden L., Wong G., Ivers N.M., Liu B., Marr S. (2018). Effectiveness of interventions for managing multiple high-burden chronic diseases in older adults: A systematic review and meta-analysis. Can. Med. Assoc. J..

[32] Bandelow B., Michaelis S. (2015). Epidemiology of anxiety disorders in the 21st century. Dialogues Clin. Neurosci..

[33] Gupta A., Eisenhauer E.A., Booth C.M. (2022). The Time Toxicity of Cancer Treatment. J. Clin. Oncol..

[34] Colin O., Labreuche J., Deguil J., Mendyk A.M., Deken V., Cordonnier C., Deplanque D., Leys D., Bordet R. (2019). Preadmission use of benzodiazepines and stroke outcomes: The Biostroke prospective cohort study. BMJ Open.

[35] Le T.T., Park S., Choi M., Wijesinha M., Khokhar B., Simoni-Wastila L. (2020). Respiratory events associated with concomitant opioid and sedative use among Medicare beneficiaries with chronic obstructive pulmonary disease. BMJ Open Respir. Res..

[36] Krishnan D., Hopman W.M., Holden R.M. (2020). Association Between Sex and Opiate and Benzodiazepine Prescription Among Patients With CKD: Research Letter. Can. J. Kidney Health Dis..

[37] Yarborough B.J.H., Stumbo S.P., Stoneburner A., Smith N., Dobscha S.K., Deyo R.A., Morasco B.J. (2019). Correlates of Benzodiazepine Use and Adverse Outcomes Among Patients with Chronic Pain Prescribed Long-term Opioid Therapy. Pain Med..

[38] Maust D.T., Petzold K., Strominger J., Kim H.M., Bohnert A.S.B. (2023). Benzodiazepine Discontinuation and Mortality Among Patients Receiving Long-Term Benzodiazepine Therapy. JAMA Netw. Open.

[39] Brunner E., Chen C.-Y.A., Klein T., Maust D., Mazer-Amirshahi M., Mecca M., Najera D., Ogbonna C., Rajneesh K.F., Roll E. (2025). Joint Clinical Practice Guideline on Benzodiazepine Tapering: Considerations When Risks Outweigh Benefits. J. Gen. Intern. Med..

