# Supplementary material for: Association Between Different Patterns of Opioid and Benzodiazepine Use and Risks of Emergency Department Visits and Hospitalizations: A Retrospective Cohort Study
Source: Healthcare (Basel). 2025 Aug 21;13(16):2073. doi: 10.3390/healthcare13162073 (PMC12386390; doi:10.3390/healthcare13162073)
Supplement: Supplementary file 1 [file healthcare-13-02073-s001.zip › healthcare-3787876-supplementary.pdf]

Supplementary material File S1 Anatomical Therapeutic Chemical [ATC] code

Table S1 Opioid Drugs Included in the Study Cohort: ATC Codes and Drug Names

ATC code	Drug
**N02AA**	Morphine HCl 10mg/tab
**N02AA**	MST(Morphine sulfate continus)30mg/f.c tab
**N02AA**	Hydromorphone PR 8mg/tab
**N02AA**	MXL(Morphine sulfate continus) 60mg/cap
**N02AA**	Morphine sulfate 15mg/tab
**N02AA**	OxyCodone Immediate Release 5mg/cap
**N02AA**	OxyCodone Controlled-Release 10mg/tab
**N02AA**	OxyCodone Controlled-Release 20mg/tab
**N02AB**	Meperidine(Pethidine) HCl 50mg/tab
**N02AB**	Fentanyl tts 25mcg/hr/patch
**N02AB**	Fentanyl tts 50mcg/hr/patch
**N02AB**	Fentanyl tts 12mcg/hr/patch
**N02AB**	Painkyl (Fentanyl buccal film) 200mcg/film
**N02AB**	Painkyl (Fentanyl buccal film) 600mcg/film
**N02AB**	Fentanyl tts 75mcg/hr/patch
**N02AB**	Fentora (Fentanyl buccal tablets) 100mcg/tab
**N02AB**	Fentora (Fentanyl buccal tablets) 200mcg/tab
**N02AE**	Buprenorphine 35 mcg/h,transdermal patch
**N02AE**	Buprenorphine 52.5 mcg/h,transdermal patch
**N02AE**	Buprenorphine 0.2mg/sublingual tab
**N02AF**	Butorphanol tartrate nasal spray 10mg/ml,2.5ml/bot
**N02AX**	Tramadol HCl 50mg/cap
**N02AX**	Ultracet tab(Tramadol 37.5mg+Acetaminophen 325mg)

Table S2 Opioid Drugs Excluded from the Study Cohort: ATC Codes and Drug Names

ATC code	Drug
**N07BC**	Buprenorphine 8mg/Naloxone 2mg sublingual tab
**N07BC**	Buprenorphine 2mg/Naloxone 0.5mg sublingual tab
**N07BC**	Methadone HCl 10mg/mL,1L/bot(oral)
**N07BC**	Methadone HCl 5mg/mL,1L/bot(oral)

Table S3. Benzodiazepine (BZD) ATC Codes and Drug Names

ATC code	Drug
**N03AE**	Clonazepam 0.5mg/tab
**N03AE**	Clonazepam 2mg/tab
**N05BA**	Clobazam 10mg/tab
**N05BA**	Alprazolam 0.5mg/tab
**N05BA**	Alprazolam S.R 0.5mg/tab
**N05BA**	Chlordiazepoxide 5mg/tab
**N05BA**	Fludiazepam 0.25mg/tab
**N05BA**	Diazepam 2mg/tab
**N05BA**	Diazepam 5mg/tab
**N05BA**	Alprazolam 0.5mg/tab
**N05BA**	LORazepam 1mg/tab
**N05CD**	Estazolam 2mg/tab
**N05CD**	Flurazepam hcl 30mg/cap
**N05CD**	Triazolam 0.25mg/tab
**N05CF**	Zolpidem 10mg/f.c tab
**N05CF**	Zolpidem CR 6.25mg/f.c tab
**N05CF**	Zolpidem 10mg/f.c tab
